# Age-specific prevalence of human papilloma virus infection among Nigerian women

**DOI:** 10.1186/1471-2458-14-656

**Published:** 2014-06-27

**Authors:** Sally N Akarolo-Anthony, Ayo O Famooto, Eileen O Dareng, Olayinka B Olaniyan, Richard Offiong, Cosette M Wheeler, Clement A Adebamowo

**Affiliations:** 1 Department of Nutrition, Harvard School of Public Health, 677 Huntington Avenue, Boston, MA 02115, USA; 2 Office of Strategic Information and Research Department, Institute of Human Virology Nigeria, 252 Herbert Macaulay Way, Abuja, Nigeria; 3 Department of Obstetrics and Gynecology, National Hospital, Abuja, Nigeria; 4 University of Abuja Teaching Hospital, Gwagwalada, Nigeria; 5 Department of Pathology, University of New Mexico Health Sciences Center, Albuquerque, NM 87131, USA; 6 Institute of Human Virology and Greenebaum Cancer Center, University of Maryland School of Medicine, Baltimore, MD 21201, USA

**Keywords:** HPV infection, Prevalence, Nigeria

## Abstract

**Background:**

Inconsistent trends in HPV prevalence by age have been described in Africa. We examined the age prevalence pattern and distribution of 37 HPV-DNA types among urban Nigerian women.

**Methods:**

The study population was a sample of 278 women who presented to cervical cancer screening programs in Abuja, Nigeria, between April and August 2012. Using a nurse administered questionnaire, information on demographic characteristics and risk factors of cervical cancer was collected and samples of cervical exfoliated cells were obtained from all participants. Roche Linear Array HPV Genotyping Test® was used to characterize prevalent HPV and log-binomial regression models were used to examine the association between potential correlates and the prevalence of HPV infection.

**Results:**

The mean age (SD) of the women enrolled was 38 (8) years. The overall prevalence of HPV was 37%. HPV 35 was the most prevalent HPV type in the study population. Among women age ≤ 30 years, 52% had HPV infection compared to 23% of those women who were older than 45 years (p = 0.006). We observed a significant linear association between age and the prevalence of HPV infections. The prevalence ratio (PR) and 95% confidence interval (CI) was 2.26 (1.17, 4.34) for any HPV infection, 3.83 (1.23, 11.94) for Group 1 HPV (definite carcinogens), and 2.19 (0.99, 4.84) for Group 2a or 2b HPV (probable or possible carcinogens) types, among women aged 18–30 years, compared to women who were older than 45 years.

**Conclusion:**

The prevalence of HPV infection was highest among younger women and decreased steadily with age among this population of urban Nigerian women.

## Background

Human papilloma viruses (HPV) are small DNA viruses, of which there are several types associated with benign and malignant conditions of the cervix, penis, vulva, vagina, anus and oropharynx [1,2]. These viruses are ubiquitous and most women in the world are probably infected with at least one type of HPV during their sexual life giving a point prevalence of 10.1% [3]. The Population Attributable Fraction of HPV associated cancer varies from 14.2% in sub-Saharan Africa and 15.5% in India to 1.2% in Australia/New Zealand and 1.6% in Northern America [2].

Previous studies have shown that the prevalence of HPV infection rises soon after the onset of sexual activity [4,5] and attains the highest value in adolescence and early adulthood after which it declines during the second and third decades of life [6]. While there seems to be a clear peak in HPV prevalence in younger women in Asia, Europe and Northern America, with a downward trend as age increases, age-specific HPV prevalence data from Central and South America appears to follow a slight U-shaped curve [7].

In Africa, the age-specific prevalence of HPV differs across countries. HPV prevalence was highest in younger women and decreased steadily with age in Kenya, Uganda and Zimbabwe [8-10] but generally reached a plateau at approximately 40 years of age in Nigeria and Mozambique [11,12]. Whereas in Senegal, South Africa and another study from rural Nigeria, the peak prevalence in young women was accompanied by a second but smaller increase in prevalence among older women [13-16]. In Gambia, HPV prevalence remained relatively constant among women aged 15 to 54 years [17]. The reason for these different age prevalence patterns in Africa remains unclear. Possible reasons include variations in sexual behavioral practices, sexual hygiene, co-morbidities, polygamy, male sexual partner behavior and male circumcision [15,16,18].

In this study, we examined the age prevalence pattern, distribution and socio-demographic correlates of HPV types among women in an urban, ethnically diverse population in Central Nigeria.

## Methods

### Study population

Between April and August 2012, we enrolled women from cervical cancer screening clinics at National Hospital, Abuja and University of Abuja Teaching Hospital, Nigeria. Abuja is the Federal Capital of Nigeria and it is located in the central part of the country. Its population is comprised of individuals from almost all ethnic groups in Nigeria, thus it is the most ethnically diverse city in the country [19]. The study participants were 18 years or older, did not have a history of hysterectomy, not pregnant and had engaged in vaginal sexual intercourse. Interviewer administered questionnaires were used to collect data on socio-demographic characteristics, sexual and reproductive histories. Samples of exfoliated cervical cells were obtained from the cervical os with a cervical brush, which was stored in Thinprep® at -80°C until processing for HPV genotyping.

### HPV detection by genotyping

HPV DNA was extracted using Gentra Puregene Cell Kit® according to the manufacturer’s instruction. HPV genotyping was done using Linear Array HPV genotyping test® (Roche Diagnostics), a qualitative in vitro test which utilizes amplification of target DNA by the Polymerase Chain Reaction and nucleic acid hybridization, and detects 37 HPV genotypes - (6, 11, 16, 18, 26, 31, 33, 35, 39, 40, 42, 45, 51, 52, 53, 54, 55, 56, 58, 59, 61, 62, 64, 66, 67, 68, 69, 70, 71, 72, 73 (MM9), 81, 82 (MM4), 83 (MM7), 84 (MM8), IS39, and CP6108. It has been validated and offers a reliable and sensitive approach for HPV DNA detection and genotyping in cervical specimens, using standardized quality-controlled reagents [20-23].

We classified HPV types according to the World Health Organization International Agency for Research on Cancer (IARC) Monographs Working Group assessment of the carcinogenicity of different HPV types [24]. HPV types were classified as follows: definite carcinogens (group 1), HPV 16, 18, 31, 33, 35, 39, 45, 51, 52, 56, 58 and 59; Probable carcinogens (group 2a), HPV 68; and Possible carcinogens (group 2b), HPV 6, 11, 26, 40, 42, 53, 54, 55, 61, 62, 64, 66, 67, 69, 70, 71, 72, 73, 81, 82, 83, 84, IS39 and CP6108.

### Statistical analysis

Descriptive analyses were performed to characterize the sampled population. Student *t*-tests were used to assess differences in the distribution of continuous variables between groups while χ^2^ and Fishers exact tests were used for categorical variables. We created age categories as follows: ≤ 30, 31 – 35, 36 – 40, 41 – 45 and > 45 years and calculated the Prevalence Ratio (PR) and 95% confidence intervals (CI) by age categories and by HPV group classification using univariate and multivariable log-binomial regression models. In separate models, we examined the association between HPV positivity and the following covariates: age, marital status, age at sexual initiation, total number of sexual partners, HIV status, contraceptive use, douching and body-mass index (BMI). To test for a linear trend, we assigned each age category the median value and modeled this variable as a continuous variable. All *P*-values were 2-sided and the analyses were performed using SAS 9.3 for UNIX statistical software.

### Ethics

The study was conducted according to the Nigerian National Code for Health Research Ethics and the Declaration of Helsinki. Ethical approval to conduct this study was obtained from the IHVN Health Research Ethics Committee and the University of Maryland, Baltimore, Institutional Review Board. Informed consent was obtained from all participants before enrollment in the study.

## Results

A total of 278 participants were enrolled in this study. We excluded 3 persons who had missing data on HPV genotype or several demographic variables, data from 275 participants were available for analysis. The mean age (SD) of the participants was 38 (8) and the median age (inter-quartile range) was 37 (31–42) years. Table 1 describes the demographic characteristics of the study participants.

**Table 1:** Characteristics of the study population

		**Age strata (years)**					
**Characteristics**	**Total**	**≤ 30**	**31 - 35**	**36 - 40**	**41 - 45**	**> 45**	**P-value**
	**n = 275**	**n = 64**	**n = 63**	**n = 57**	**n = 56**	**n = 35**	
	**Median (Inter-quartile range)**						
**Age (years)**	37 (31–42)	28 (26–29)	33 (32–35)	38 (37–39)	43 (42–44)	49 (47–53)	
**Age at sexual initiation**	19 (18–22)	19 (17–21)	20 (18–23)	19 (17–22)	20 (18–22)	19 (18–21)	<0.001
**Total sex partners**	3 (1–4)	2 (1–4)	3 (2–5)	3 (1–3)	2 (1–5)	2 (1–3)	<0.001
**BMI (kg/m**^**2**^**)**	27 (22–31)	22 (19-27	25 (22–29)	27 (24–32)	29 (25–33)	31 (29–36)	<0.001
	**n (Percentage)**						
**Education**							0.15
- Primary	38 (14)	11 (17)	8 (13)	10 (17)	3 (5)	6 (17)	
- Secondary	191 (69)	49 (77)	42 (67)	38 (67)	39 (70)	23 (66)	
- Tertiary	46 (17)	4 (6)	13 (20)	9 (16)	14 (25)	6 (17)	
**Marital status**							0.41
- Married	169 (61)	34 (53)	38 (60)	40 (70)	36 (64)	21 (60)	
- Not married	106 (39)	30 (47)	25 (40)	17 (30)	20 (36)	14 (40)	
**Contraceptive use**							0.84
- Yes	103 (38)	24 (38)	20 (32)	22 (39)	22 (39)	15 (43)	
- No	172 (62)	40 (62)	43 (68)	35 (61)	34 (61)	20 (57)	
**Condom use**^**#**^							0.36
- Yes	3 (1)	2 (3)	0 (0)	1 (2)	0 (0)	0 (0)	
- No	272 (99)	30 (97)	63 (100)	56 (98)	56 (100)	35 (100)	
**Douching**							0.22
- Yes	174 (63)	39 (61)	46 (73)	31 (54)	38 (68)	20 (57)	
- No	101 (37)	25 (39)	17 (27)	26 (46)	18 (32)	15 (43)	
**HIV infection**							0.19
- Positive	148 (58)	35 (59)	40 (69)	30 (53)	25 (47)	18 (56)	
- Negative	111 (42)	24 (41)	18 (31)	27 (47)	28 (53)	14 (44)	
**Any HPV infection**							0.006
- Positive	101 (37)	33 (52)	27 (43)	20 (35)	13 (23)	8 (23)	
- Negative	174 (63)	33 (48)	36 (57)	37 (65)	43 (77)	27 (77)	
**Multiple HPV infection**							0.001
- Positive	46 (17)	16 (25)	18 (29)	7 (12)	3 (5)	2 (6)	
- Negative	229 (83)	48 (75)	45 (71)	50 (88)	53 (95)	33 (94)	
**Group 1 HPV infection**							0.03
- Positive	61 (22)	21 (33)	17 (27)	12 (21)	8 (14)	3 (9)	
- Negative	214 (78)	43 (67)	46 (73)	45 (79)	48 (86)	32 (91)	
**Group 2a HPV infection**							0.18
- Positive	8 (3)	4 (6)	3 (5)	1 (2)	0 (0)	0 (0)	
- Negative	267 (97)	60 (94)	60 (95)	56 (98)	56 (100)	35 (10)	
**Group 2b HPV infection**							
- Positive	67 (24)	24 (38)	20 (32)	11 (19)	6 (11)	6 (17)	0.004
- Negative	208 (76)	40 (62)	43 (68)	46 (81)	50 (89)	29 (83)	

^#^Consistent condom use in the past 2 years.

The prevalence of any of the 37 HPV types in the study population was 37% (101/275). Group 1 HPV types (definite carcinogens) were found in 22% (61/275) of the study participants. HPV 35, a group 1 type, was the most prevalent type of HPV found in this study, it was found in 5% (14/275) of the women studied. Other group 1 HPV types found among the women studied were HPV 56, 58, 45 and 59 in decreasing order of prevalence. The prevalence of HPV16 and 18 in this population was low; HPV 16 was found in 2% (6/275) and HPV 18 was found in 2.5% (7/275) of the women studied. The prevalence of group 2a (Probable carcinogens) HPV type in the study participants (HPV68) was 3% (8/275). Group 2b HPV types (Possible carcinogens) were found in 24% (67/275) of the study participants. HPV81 and HPV42 were the most prevalent group 2b types, each was found in 4.7% (13/275) of the women studied.

**Figure 1:**
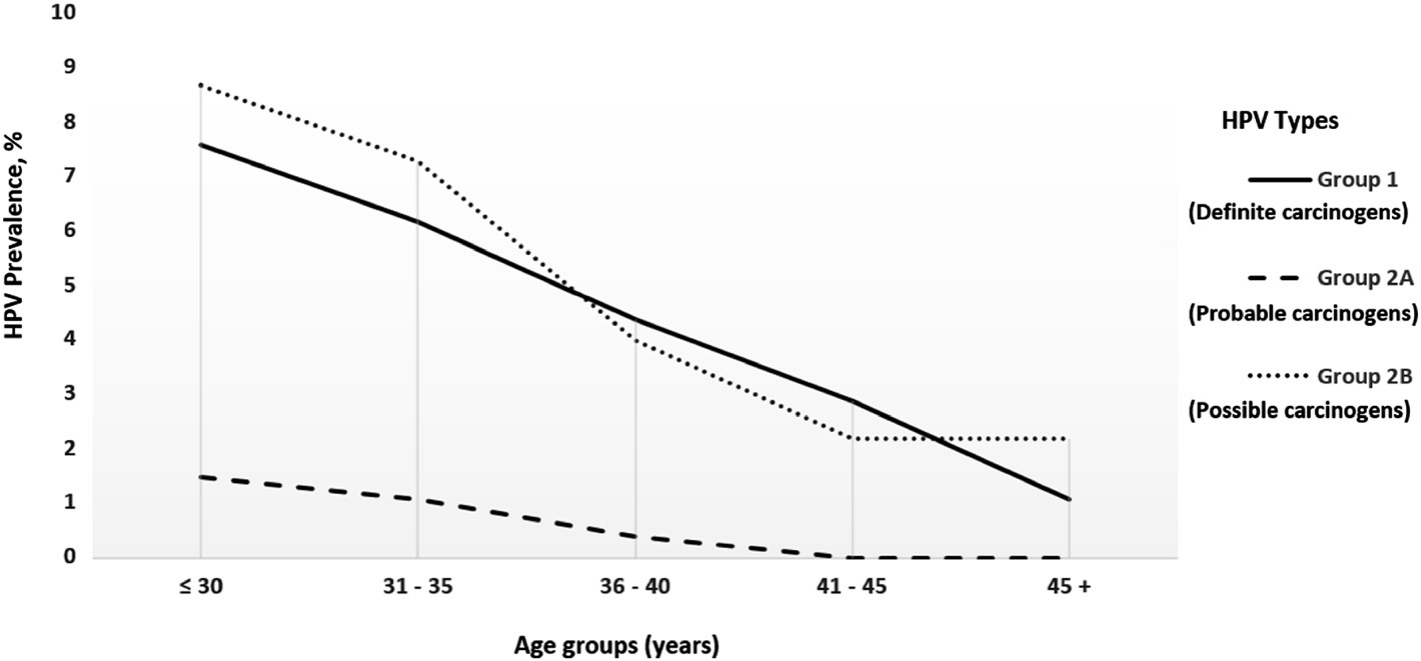
Prevalence curves of HPV types, by age group.

Among women who had HPV infection, the age group distribution was 32% (33/101) for ≤30 years; 27% (27/101) for 31 – 35 years, 20% (20/101) for age group 36 – 40 years, 13% (13/101) for 41 – 45 years and 8% (8/101) for women older than 45 years (Table 1). The prevalence curves of HPV types for each age group are shown in Figure 1. The prevalence of any HPV infection was significantly reduced with increasing age. The multivariable PR of any HPV infection was 2.26 (1.17 to 4.34) for the lowest age category compared with the highest age category (referent group). This association remained significant for definite carcinogenic and probable/possible carcinogenic HPV types.

We found multiple HPV infections in 12.7% (35/275) of the women. Multiple HPV infections were more prevalent among younger women. Compared to women in the highest age group (>45 years), the PR (95% CI) of multiple HPV infection was 4.38 (1.07, 17.94), 5.00 (1.23, 20.30), 2.15 (0.47, 9.77) and 0.94 (0.16, 5.33), p for linear trend = 0.0004, for age group ≤ 30, 31 – 35, 36 – 40 and 41 – 45, respectively (Table 2).

**Table 2:** HPV prevalence ratios by age strata and carcinogenic risk

**HPV Types**	**Age strata (Years)**										***P *** **for Trend**
	**≤ 30**		**31 - 35**		**36 - 40**		**41 - 45**		**> 45**		
	**N**	**PR (95% CI)**	**N**	**PR (95% CI)**	**N**	**PR (95% CI)**	**N**	**PR (95% CI)**	**N**	**Ref**	
Any HPV (n = 136)	49	2.26 (1.17, 4.34)	40	1.88 (0.96, 3.67)	24	1.54 (0.76, 3.10)	14	1.02 (0.47, 2.20)	9	1.00	0.0003
Multiple HPV (n = 46)	16	4.38 (1.07, 17.94)	18	5.00 (1.23, 20.30)	7	2.15 (0.47, 9.77)	3	0.94 (0.16, 5.33)	2	1.00	0.0004
Group 1 (n = 61)	21	3.83 (1.23, 11.94)	17	3.15 (0.99, 10.00)	12	2.46 (0.74, 8.10)	8	1.67 (0.47, 5.86)	3	1.00	0.0014
Group 2* (n = 75)	28	2.19 (0.99, 4.84)	23	1.85 (0.82, 4.18)	12	1.13 (0.46, 2.77)	6	0.62 (0.22, 1.79)	6	1.00	0.0007

*Group 2 = Group 2A and 2B.

The PR (95% CI) of any HPV infection was 2.54 (1.69, 3.81) p = < 0.001, comparing women who were HIV positive with those who were HIV negative. In multivariable analysis, we found a significant association between HIV and prevalent HPV infection, the PR (95% CI) of HIV for any HPV infection was 2.27 (1.48 to 3.48). HIV status was also associated with the prevalence of group 1 and group 2 HPV types in multivariable analysis, adjusting for age, marital status, age at sexual initiation, number of sexual partners, use of contraceptives, douching and body mass index. The multivariable adjusted PR (95% CI) was 4.01 (1.96, 8.21) for HIV and group 1 HPV, and 1.87 (1.11 to 3.17) for HIV and group 2 HPV (Table 3).

**Table 3:** Univariate and multivariable prevalence ratios of potential correlates of HPV positivity

**Potential correlates**	**Any HPV**		**Group 1**		Group 2*	
	**PR (95% CI)**		**PR (95% CI)**		**PR (95% CI)**	
	**Univariate**	**Multivariable**	**Univariate**	**Multivariable**	**Univariate**	**Multivariable**
Age	0.96 (0.94, 0.98)	0.98 (0.95, 1.00)	0.95 (0.92, 0.98)	0.96 (0.93, 1.00)	0.95 (0.92, 0.98)	0.97 (0.94, 1.00)
Marital status	1.17 (0.85, 1.59)	0.99 (0.74, 1.32)	1.43 (0.92, 2.22)	1.25 (0.80, 1.95)	1.28 (0.84, 1.94)	1.09 (0.70, 1.68)
Age at sexual initiation	0.98 (0.94, 1.03)	1.00 (0.95, 1.05)	0.96 (0.90, 1.03)	0.98 (0.71, 1.36)	0.98 (0.93, 1.04)	1.00 (0.78, 1.28)
Number of sexual partners	0.99 (0.94, 1.05)	1.01 (0.95, 1.07)	1.02 (0.95, 1.10)	1.04 (0.96, 1.13)	1.00 (0.93, 1.08)	1.01 (0.93, 1.09)
HIV status	2.54 (1.69, 3.81)	2.27 (1.48, 3.48)	3.68 (1.95, 6.92)	4.01 (1.96, 8.21)	2.21 (1.32, 3.67)	1.87 (1.11, 3.17)
Contraceptive use	1.20 (0.86, 1.67)	1.24 (0.89, 1.73)	1.16 (0.72, 1.85)	0.98 (0.60, 1.60)	1.24 (0.79, 1.94)	1.34 (0.83, 2.17)
Douching	0.95 (0.69, 1.32)	1.06 (0.78, 1.44)	1.11 (0.71, 1.75)	1.54 (0.94, 2.52)	0.90 (0.58, 1.39)	1.05 (0.67, 1.66)
Body mass index	0.94 (0.90, 0.97)	0.96 (0.93, 1.00)	0.93 (0.88, 0.98)	0.97 (0.91, 1.02)	0.96 (0.91, 1.01)	0.97 (0.92, 1.03)

*Group 2A and 2B.

The multivariable PR (95% CI) of any HPV infection was 0.96 (0.93 to 1.00), comparing women with BMI ≥ 25 kg/m^2^ to those with BMI 18.5 - < 25 kg/m^2^. There was no significant association between BMI and group 1 or group 2 HPV infections. The multivariable PR (95% CI) was 0.97 (0.91, 1.02) for BMI and group 1; and 0.97 (0.92, 1.03) for BMI and group 2 HPV infections, comparing women with BMI ≥ 25 kg/m^2^ to those with BMI 18.5 - < 25 kg/m^2^.

## Discussion

In this cross-sectional study of urban Nigerian women, the prevalence of HPV infection was 37%. We observed a significant inverse linear association between age and the prevalence of HPV infections. This prevalence was approximately 4 times higher in women aged 18 – 30 years compared to women who were older than 45 years and younger women were more likely to have carcinogenic HPV types.

The prevalence of HPV infection among women in our study population was higher than had been reported in previous studies from Nigeria. Among 932 women surveyed between 1999 and 2000 in southwestern Nigeria, the prevalence of HPV was 26.3% [25]. Our finding of HPV35 as the most prevalent type is similar to findings from southwest Nigeria and southern Mozambique, among women without invasive cervical cancer [12,25].

Different trends have been reported on the age prevalence of HPV in Africa. The results of some studies showed there was a high peak of HPV prevalence among younger women which decreased with age, but among older women the prevalence either decreased [8,9], reached a plateau [12,26] or increased [13,14]; whereas one study showed a relatively constant prevalence across all ages [17]. Studies in rural and semi-rural southwestern Nigeria showed that the age prevalence of HPV was highest in younger women, declined with age and reached a plateau [11] or increased [15] among older women. Although the variation in age prevalence of HPV is well documented and appears to largely reflect differences in sexual behavior across geographical regions [7], the difference between our result and that of the previous studies from Nigeria may reflect characteristics of the populations surveyed. We studied an urban population of women in Abuja, central Nigeria who presented to hospitals for cervical cancer screening, while these other studies were community surveys in which participants were visited at their homes in southwestern Nigeria and asked to participate.

The results of some studies suggests that HIV infection is a known risk factor for HPV infection [27]. In our study, we found an association between HIV and HPV infection and this association remained statistically significant when we examined the association between HIV and different categories of HPV. The results of our analyses on HIV and high-risk HPV infections among Nigerian women have been previously described [28]. Although the prevalence of HPV infection appeared to be associated with some socio-demographic factors, these results did not reach significant levels in our multivariable analysis.

We found a marginal inverse association between any HPV infection and BMI in univariate analysis but this association was null in multivariable analysis. Prior studies of the association between body weight and HPV infections have yielded conflicting results. Among female sexual workers in Spain, there was an inverse association between high-risk HPV prevalence and BMI. In contrast, a study based on the US National Health and Nutrition Examination Survey (NHANES) showed that BMI was not associated with prevalence of HPV [29]. The relationship between BMI and the risk of HPV infection needs further evaluation.

Our study has several important limitations. First, the sample size was small hence we were unable to examine the prevalence of HPV infection among women age 60 years and above. We are therefore unable to determine whether the prevalence of HPV in this study population would have continued to fall, remained stable or risen. Second, because of the cross sectional design of our study we could not examine temporal trends of potential correlates of HPV and the risk factors for persistent HPV infections. With continued follow up of this population and repeated sampling, we will be able to report the pattern and spectrum of persistent HPV infection in Nigeria in future.

## Conclusion

The age prevalence pattern of HPV infection among women in cervical cancer screening programs in Nigeria is similar to that in many developed countries. Replication of this study in other urban African populations is required. Further studies to investigate epidemiological correlates of persistent high risk HPV infection are also needed. These studies will be useful in providing evidence for policies relating to the use of HPV DNA tests for screening, utility and coverage of current HPV vaccines and allocation of resources for HPV infections and cervical cancer prevention and treatment.

### Study design

Cross-sectional survey.

## Abbreviations

BMI: Body mass index; CI: Confidence interval; CIN: Cervical intraepithelial neoplasm; HIV: Human immunodeficiency virus; HPV: Human papillomavirus; IARC: International Agency for Cancer Research; IHVN: Institute of Human Virology Nigeria; NHANES: National Health and Nutrition Examination Survey; PCR: Polymerase Chain Reaction REDCap Research Electronic Data Capture; PR: Prevalence ratio; SD: Standard deviation.

## Competing interests

CMW has received through the University of New Mexico, funds from grants and cooperative agreements from the US National institutes of Health related to cervical screening, funds from GSK for HPV vaccine studies and reimbursements for travel related to publication activities and equipment and reagents from Roche Molecular Systems for HPV genotyping.

Other authors report no competing interest.

## Authors’ contributions

SNA analyzed the data and drafted the manuscript. AOF, EOD, MA and IL coordinated the study and managed data accrual. OO and RU, site investigators, contributed to the study design, implementation and provided revisions of the manuscript. CMW performed HPV genotyping, contributed to data interpretation and provided revisions of the manuscript. CAA conceived the study, obtained funds, contributed to the study design and provided critical revisions of the manuscript. All authors read and approved the final manuscript.

## Pre-publication history

The pre-publication history for this paper can be accessed here:

http://www.biomedcentral.com/1471-2458/14/656/prepub
